# Insights into the Structure and Energy of DNA Nanoassemblies §

**DOI:** 10.3390/molecules25235466

**Published:** 2020-11-24

**Authors:** Andreas Jaekel, Pascal Lill, Stephen Whitelam, Barbara Saccà

**Affiliations:** 1 Zentrum für Medizinische Biotechnologi (ZMB), University of Duisburg-Essen, 45141 Essen, Germany; andreas.jaekel@uni-due.de; 2 Structural Biochemistry, Max Planck Institute of Molecular Physiology, 44227 Dortmund, Germany; pascal.lill@mpi-dortmund.mpg.de; 3 Molecular Foundry, Lawrence Berkeley National Laboratory, Berkeley, CA 94720, USA; swhitelam@lbl.gov

**Keywords:** DNA self-assembly, thermal annealing, DNA origami, nucleation, energy landscape

## Abstract

Since the pioneering work of Ned Seeman in the early 1980s, the use of the DNA molecule as a construction material experienced a rapid growth and led to the establishment of a new field of science, nowadays called structural DNA nanotechnology. Here, the self-recognition properties of DNA are employed to build micrometer-large molecular objects with nanometer-sized features, thus bridging the nano- to the microscopic world in a programmable fashion. Distinct design strategies and experimental procedures have been developed over the years, enabling the realization of extremely sophisticated structures with a level of control that approaches that of natural macromolecular assemblies. Nevertheless, our understanding of the building process, i.e., what defines the route that goes from the initial mixture of DNA strands to the final intertwined superstructure, is, in some cases, still limited. In this review, we describe the main structural and energetic features of DNA nanoconstructs, from the simple Holliday junction to more complicated DNA architectures, and present the theoretical frameworks that have been formulated until now to explain their self-assembly. Deeper insights into the underlying principles of DNA self-assembly may certainly help us to overcome current experimental challenges and foster the development of original strategies inspired to dissipative and evolutive assembly processes occurring in nature.

## 1. Introduction

The discovery of the double-helical structure almost 70 years ago [1] is universally recognized as one of the pivotal points in the history of science. Since then, the DNA molecule has shown us its preeminent role in life, bridging the molecular level (i.e., the DNA and its protein products) with the system level of the cell and entire organism [2]. Most surprisingly, this task is accomplished through a simple four-letters code and one base-pair rule that provides DNA with a unique molecular feature—namely, self-recognition. In other words, the DNA carries in its structure the information for its own self-assembly, such that, given one strand, the complementary strand needed to form the double helix is uniquely defined. In the early 1980s, Ned Seeman recognized the tremendous implications of such a simple property for construction purposes and demonstrated how single-stranded DNA sequences can be programmed to associate into ordered molecular objects in a fully predictable fashion [3]. This led to the foundations of a new field of science, later known as structural DNA nanotechnology [4]. A notable breakthrough in the field occurred in 2006, when Paul Rothemund published a new method for folding DNA, called scaffolded DNA origami [5]. Despite relying on the same Watson-Crick base pairing rule, the DNA origami technique uses a long single-stranded DNA chain for the formation of the target shape and, by doing so, efficiently reduces the probability for spurious structures to form (we will see this process more in detail later). The consequence of this new building principle has been a dramatic improvement in our ability to engineer DNA nanomaterials, enabling us to gain access to structures with a level of sophistication previously unimaginable [6,7]. This, together with the availability of user-friendly design tools and the synthetic availability of oligonucleotides at relatively low costs, prompted the introduction of DNA nanotechnology into about 400 laboratories around the world and transformed a DNA-based building strategy into one of the methods of choice for controlling matter distribution with sub-nanometer precision [8,9,10].

Structural DNA nanotechnology relies on precise design rules. Thus, a deep understanding of the relationship between the topology of the DNA molecule and its mechano-elastic and thermal properties is necessary to construct objects that are more sophisticated than a canonical double helix. Not surprisingly, the pioneer of the field is a crystallographer, and his initial works on DNA branched motifs can be best appreciated and understood only if viewed in terms of DNA topology and related energetic stability. Fortunately, modern computer-aided programs have facilitated the accessibility of DNA design approaches to nonexperts; nevertheless, any effort devoted to the improvement of current strategies or the creation of new ones necessarily requires awareness of the underlying theoretical frameworks.

Our purpose, in this review, is to give an overview of such theoretical background. We will therefore report in some detail the structural and energetic properties of DNA nanostructures and present the current hypotheses and models of the mechanisms that regulate their self-assembly. We will start by describing the DNA duplex (Section 2) and will move to the basic building block of all DNA nanostructures—namely, the Holliday Junction (HJ), also called four-arm junction (Section 3). We will survey both the structural and energetic properties of this fundamental motif and summarize the final view that has been acquired through numerous thermodynamic and kinetic studies, both in bulk and at the single-molecule level. In Section 4, we will briefly review more complex DNA branched motifs, thus laying the ground for a better understanding of the self-assembly approach originally developed by Seeman, later called the multi-stranded (or tile-based) approach. We will then focus on the other main design school, i.e., the scaffold-based (or DNA origami) approach, and briefly discuss the structural and thermal properties of such objects (Section 5). Finally, in Section 6, we will present and critically compare both design strategies and discuss their analogy to the nucleation-and-growth mechanism of crystal formation. We will conclude by reporting recent examples of alternative approaches for guiding DNA self-assembly. In such methods, structural stability and dynamic reconfiguration properties are cleverly combined to enable the system to read information from the external environment and respond to a change, thus adapting to a new condition. These strategies, although still in their infancy, pave the way to a completely new paradigm of DNA self-assembly that—by mimicking nature—allows the system to undergo selection and, in this sense, to evolve.

## 2. The Double Helix

**Figure 1:**
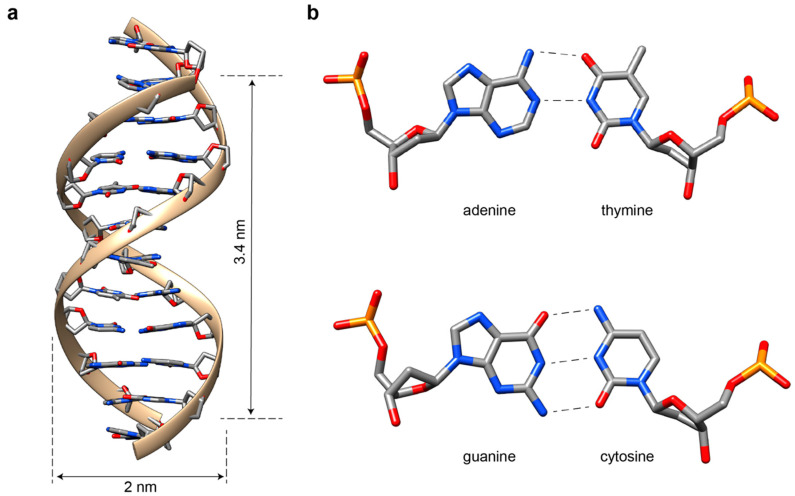
The DNA helix. (**a**) The DNA molecule in its most common B-form is a right-handed helical structure, which is 2-nm-wide and rises ca. 3.4 nm every helical turn (corresponding to ca. 10.5 base pairs). The four nucleobases (A, G, T and C) and their paring rule are also indicated (**b**). Note that, whereas AT pairs are stabilized by two hydrogen bonds, GC pairs involve three bonds, which explains their higher contribution to helical stabilization. Figure modified from open sources (Protein Data Bank (PDB): 1DUF).

A single-stranded DNA is a polynucleotide chain consisting of the nitrogen-containing nucleobases adenine (A), guanine (G), thymine (T) and cytosine (C), covalently linked to one another through a sugar-phosphate backbone. In its most common B-form, the DNA molecule is composed of two such strands oriented in an antiparallel fashion and wrapped together into a right-handed double helix, which is about 2-nm-wide and has a helical pitch of 3.4 nm every 10.5 base pairs (bp) (Figure 1). Two main forces are responsible for formation of the double helix: (i) stacking among the aromatic rings of adjacent nucleobases along each strand and (ii) hydrogen bonding between the bases of opposite strands. Whereas the former mostly contributes to the stabilization of the structure, the latter is what drives the self-recognition process according to the Watson-Crick complementarity rule (A/T and G/C). Base pairing is therefore the feature that makes the DNA molecule an ideal semanto-morphic material, i.e., a material that holds the information (semantic) for building its own structure (morphology). Most artificial DNA nanostructures, from the immobile Holliday junction to GDa-sized DNA origami multimers and DNA bricks, are based on the association of DNA oligonucleotides into double helical segments and loops intertwined with one another in various ways. The use of alternative double helical forms (such as Z-DNA) has been limited to few examples, most likely due to the requirement of non-physiological conditions such as reduced water activity or the presence of metal-ion complexes [11,12]. Other DNA secondary structures, such as G-quadruplexes, i-motifs, hairpin dumbbells or loops, have been instead typically used as chemical sensors or topological markers. 

When considering the self-assembly of a DNA based object, the most fundamental process, we have to deal with is the formation of a DNA duplex. The hybridization of two complementary strands into a full duplex has been investigated for several decades, and, although we have a thorough understanding of much of this process, some aspects still remain unsolved (for a comprehensive review on nuclei acid hybridization reactions, see reference [13]). On the basis of merely thermodynamic arguments, the annealing process is typically described as a two-state or an all-or-none model, i.e., as an equilibrium between the completely dissociated form (the two separated strands) and the fully associated form (the duplex). The nearest-neighbor model [14] can be applied to calculate the free-energy change and equilibrium constant of the association/dissociation reaction, and computational tools have been developed to estimate the rate coefficients for both the forward and reverse step [15,16]. Thus, in principle, we hold all necessary knowledge to describe and predict the outcome of a DNA duplex once the sequences of the interacting strands are given. This simplification greatly helps in the analysis and interpretation of experimental data but is inadequate to explain the strong deviations from ideality that have been observed in more complex systems. For example, as recently reported by Wales and coauthors [17], a six-nucleotide-long sequence might present a level of complexity that cannot be easily recapitulated by a two-state model and requires the introduction of sequence-specific parameters, such as modulators of DNA-hybridization mechanisms [18]. The general picture is that of a global downhill process that starts with the formation of a critical nucleation seed of few base pairs and proceeds with the propagation of the remaining base pairing interactions according to a zippering or slithering mechanism [19]. The path to the target state might be, however, rather “frustrated”, meaning that the profile of the free-energy landscape presents significant hills and valleys that may cause metastable intermediates to become kinetically trapped. Thus, despite the fact that the native Watson-Crick duplex is often the conformational state corresponding to the absolute minimum of energy, local minima can be significantly populated in some cases.

The probability of visiting those metastable states, i.e., the kinetic route travelled by the system, is strongly dictated by the sequence content and length of the interacting strands and by the experimental conditions used. For example, dissociation rate coefficients may span more than eight orders of magnitude when going from a 10-mer to a 20-mer [20], whereas GG tracts have been found to hybridize much more rapidly than GC tracts of the same length [17]. This fact indicates that, in addition to the total GC content, the order of nucleobases along the sequence (i.e., the entropic contribution to the system) may greatly affect the thermodynamic stability of competing conformational ensembles and, thus, determine the preferred hybridization pathway. The energy landscape of DNA duplex formation can be also shaped by the presence of metal ions in solution, whose differential affinity towards the phosphate or nucleobases of the double helix essentially affects the capability of the complementary strands to approach and mutually interact [21]. Typically, Mg(II) ions are used in the form of chloride or acetate salts for the preparation of DNA self-assembly buffers. The high affinity of magnesium ions for the phosphate backbone of the DNA is supposed to provide sufficient Debye screening to enable the winding of DNA chains into compact structures, with bending and torsional stiffness dependent on salt concentration [22]. Thus, although the DNA duplex can be considered a well-known reference structure for most of our nanotechnological applications, with predictable thermodynamic and kinetic properties, it is worth noting that, when analyzed in detail, even this familiar system may still present unusual and peculiar features. 

## 3. The Four-Arm Junction 

**Figure 2:**
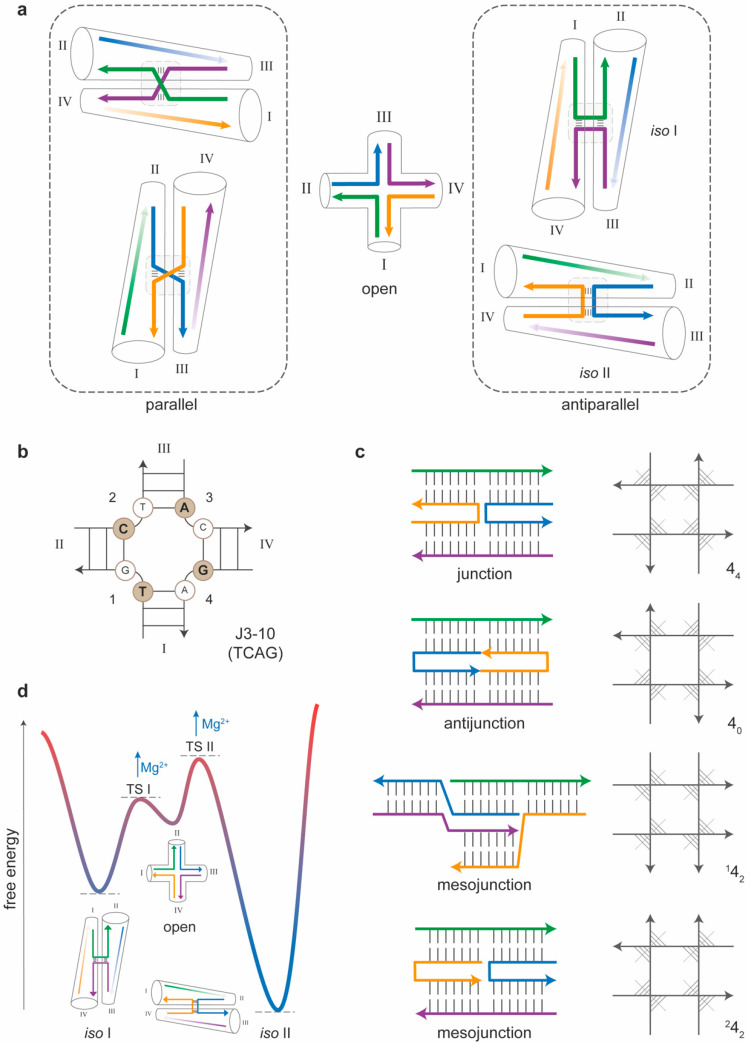
The four-arm junction. (**a**) An immobile four-arm junction can exist in four conformational states, depending on the orientation of the paired helices and the stacked base pairs at the crossover. (**b**) According to Altona’s rules, the J1 motif is classified as J3-10 and is identified by the quartet of nucleobases that face the crossover at the 5′ end of each strand (here, TCAG). (**c**) Besides the canonical junction, a four-strand branched molecule can form an antijunction or one of two mesojunctions. The topology of each four-arm junction can be schematically represented by four strands flanking a central square, with the orientation of the helical axes being either radial (4_4_), tangential (4_0_) or mixed (^1^4_2_ or ^2^4_2_). (**d**) Thermodynamic and kinetic studies on HJs enabled to trace the energy landscape that describes the folding of the motif and its conformational transition between two stable states (*iso* I or *iso* II), passing through a less stable intermediate open form.

To enable the growing of a DNA nanostructure beyond a simple duplex, single-stranded stretches must be shared among at least two distinct helices, thus leading to formation of DNA branched motifs [23]. Inspired by the naturally occurring Holliday junction, a key intermediate in many types of genetic recombination and double-strand break repair mechanisms, Seeman created the first immobile four-arm junction (called J1), which signed the beginning of structural DNA nanotechnology [3]. The four-arm junction is the fundamental building block of all DNA nanostructures (Figure 2a). A full comprehension of its structural and energetic properties is therefore essential to analyze the behavior of more complex assemblies and understand their mechanism of formation. In the following, we will survey the structural features of the four-arm junction and describe how these are affected by the base sequence of its component strands and the way they are connected. We will then discuss the thermodynamic properties of four-arm junctions and the kinetics of post-assembly isomerization, which, together, summarize our current view of the energy landscape that describes the folding of this motif and its conformational transition between two distinct states. 

### 3.1. Sequence-Dependent Properties of Four-Arm Junctions

The naturally occurring Holliday junction forms when two homologous double-stranded (ds) DNA molecules exchange their strands at a common branch point [24,25]. The main feature of this natural motif is therefore the sequence symmetry of its component strands, which causes the branch point to migrate. Although this feature is essential for the successful accomplishment of the biological function, it is of course deleterious for most construction purposes. A nonmigrating branch point can be achieved by minimizing the sequence symmetry of the system. The junction becomes thus immobile, in the sense that its topological isomerization into alternative motifs is prevented. An immobile four-arm junction can nevertheless assume four possible conformations, linked through the open form of the motif (Figure 2a). In the stacked conformations, the relative orientation of the two intersected helical domains is either parallel or antiparallel, and, for each of these orientations, either one or the other of the two possible tetrads of nucleobases at the crossover is involved in stacking interactions (Figure 2a). Antiparallel isomers are largely favored with respect to their parallel analogs, restricting the number of realistically attainable structures to two. On the other hand, which of the two antiparallel stacked isoforms (referred to as *iso* I and *iso* II) will preferentially form in solution is largely dependent on the sequence of the nucleobases at the crossover and in its direct vicinity in a way that remains still unclear. After the first J1 junction from Seeman, other four-way motifs have been realized and analyzed for their conformational preference and stability. The wealth of data obtained and the different schemes used to represent the structures called for a systematic classification of synthetic HJs. Altona et al. [26] proposed a set of graphical and base priority rules for the unambiguous representation of DNA junctions. The application of these rules to a four-arm junction leads to a final scheme in which the open form is drawn with the 5’ terminus of strand 1 at the bottom left side of the motif, and the remaining strands are placed in a clockwise fashion terminating with the 3′ end of strand 4 on the bottom right side of the structure (Figure 2b). Accordingly, the four helical portions of the junction (indicated as I, II, III and IV) can fold either in the I/IV + II/III or I/II + III/IV coaxial stacking conformer (corresponding to the *iso* I and *iso* II species, respectively; see Figure 2a). This classification resulted in a total of 70 four-arm junctions with a unique crossover sequence (36 of which are immobile), further divided into six classes, depending on the quartet of permutated purine/pyrimidine bases at the center of the motif. According to this nomenclature, junction J1 is classified as J3-10 and displays the unique sequence TCAG at the crossover (Figure 2b). Gel electrophoresis analysis [27] and fluorescence resonance energy transfer (FRET) experiments [28] suggested that the J1 motif is mostly in the *iso* I conformation. A different junction, initially called J2 and characterized by the central sequence CGTA (classified as J4-2), displays instead an inverted order of stability of the two conformers with a strong preference for *iso* II. On the other hand, junction J5-4 (CTTG) shows no preference at all.

### 3.2. Topology-Dependent Properties of Four-Arm Junctions

As mentioned above, DNA junctions are composed of multiple strands with each strand participating in (at least) two different double helices. Relatively early, Seeman and coworkers realized that junctions are only one member of a larger class of double-helical multi-stranded molecules, which include antijunctions and mesojunctions [29]. Accordingly, a four-strand complex may fold into a (canonical) junction, an antijunction or one of two types of mesojunctions (Figure 2c, left panel). A convenient way to distinguish these molecules relies on drawing the four helices as straight lines so that they flank a central square (Figure 2c, right panel). The orientation of each strand is indicated by an arrowhead at the 3′ end, while the helical axes are depicted as segments that cut the angle between two strands of opposite polarity. In the conventional four-arm junction, the four axes are pointing radially and meet in the center of the square. This motif is indicated as 4_4_, where the first digit indicates the number of strands composing the motif and the subscript designates the number of helices that are radial. When, instead, all helical domains meet outside the square, i.e., their directions are circumferential rather than radial; the resulting motif is termed an antijunction and is indicated as 4_0_. Two additional motifs can be created, in which a pair of radial helical axes and a pair of tangential helical axes coexist; however, they are either alternatively or adjacently arranged. These motifs, termed mesojunctions, are respectively indicated as ^1^4_2_ and ^2^4_2_. Of all four possible four-arm branched motifs, the conventional 4_4_ junction shows the highest stability [29] and is, therefore, the most suitable building block for the construction of extended DNA architectures. 

### 3.3. Thermal Stability and Isomerization of Four-Arm Junctions

Several studies have been performed to characterize the thermodynamic and kinetic properties of synthetic analogs of the Holliday junction, leading to a detailed view of the energy landscape that describes the self-assembly process of the motif (i.e., its thermally driven folding/unfolding) and its post-assembly structural reconfiguration (i.e., the *iso* I/*iso* II isomerization) (Figure 2d). Initial investigations on the thermal stability of four-way junctions were performed early after the development of the first immobile motif [30]. In their work, Marky et al. applied UV spectroscopy and differential scanning calorimetry to determine the enthalpy of transition of the J1 motif in a model-dependent and model-independent fashion. These values were then compared with the enthalpy of transition associated to the individual duplex arms to evaluate the impact of the central connection on the relative stability of the entire motif. The data show that each individual arm, as well as the J1 junction, exhibits a two-state melting behavior and that the enthalpy of formation for the full motif (ca. 190 kcal/mol) is equal to the sum of the enthalpies of the single arms (each one is about 48 kcal/mol). This implies that the geometry at the crossroads of the junction does not lead to disruption of enthalpically favorable interactions and, in fact, has been shown to preserve the conformation of the isolated duplex arms. Importantly, this conclusion does not mean that the formation of the junction has no enthalpic cost (as explained below, it is instead the result of positive and negative contributions that compensate each other). Several structural studies, particularly from Lilley’s group [28,31], have contributed to solving the in-solution structure of the four-way junction in different environmental conditions, portraying the full energy landscape for folding and post-assembly reconfiguration of this motif. Applying a fluorescence resonance energy transfer (FRET), they determined the relative distances between the ends of the junction and, therefore, the path of the strands, revealing a right-handed antiparallel X-shaped structure. Global comparison of the FRET efficiencies obtained by labeling many identical DNA molecules at different terminal positions allowed to individuate the isomeric form assumed by a defined set of sequences and how this conformation varied in the presence of added metal ions. The results show that the transition from the low-salt extended form to the stacked X-structure occurs in a continuous noncooperative manner as the concentration of the ion (Na^+^, Mg^2+^ or Ca^2+^) increases. The role played by the cations is thus to supply a high local density of highly mobile positive charges that screen the negative charges around the phosphates, eventually allowing the compact junction to form. Molecular dynamics simulations have shown that, in divalent electrolytes, Mg^2+^ ions act as a bridge between two minor grooves of aligned DNA molecules, with an attractive force of up to 42 pN per one turn of a DNA helix [32]. The final view is therefore the following: the free energy for folding a four-way junction is largely determined by two opposite contributions: favorable helix-to-helix stacking and unfavorable electrostatic interactions at the crossing point of the helices. Whereas the former interactions determine the isomeric form of the structure (in a way that remains unclear), the latter are reduced by the presence of cations, which are then crucial to the stable assembly of the motif.

Besides the thermal stability, the kinetics of *iso* I to *iso* II isomerization has also been a matter of intense investigation. However, conventional kinetic studies in bulk solution do not permit the full characterization of the conformational transition, due to the inability to synchronize the junction into a single conformer. This challenge was successfully overcome by Ha and coworkers, as they employed single-molecule FRET to detect the conformational transition of two individual junctions, one heavily biased towards the stacking conformer *iso* II (with a central sequence CGTA, classified as J4-2) and the other with nearly equal populations of each conformational state (central sequence CCGG, classified as J3-4) [33]. The data reveal that the transition rates between the two conformers are sequence-dependent (with rate coefficients about 10 s^−1^ at a 1 pN applied load) and significantly decrease with an increased magnesium concentration while maintaining a 1:1 ratio. This implies that (i) magnesium ions directly affect the energy barrier between the two conformers but not their relative stability and (ii) that the open structure, which is stable in the absence of magnesium ions, is a necessary transient species (Figure 2d). Later on, the same group applied single-molecule force measurements to identify the extent of the molecular forces involved in the conformational transition, finding values in the order of 1 pN [34]. The final result was the full mapping of the energy landscape and the identification of the transition state structures that enable the passage from the open form to one of the two stacked isomers. 

## 4. The Multi-Stranded Approach

**Figure 3:**
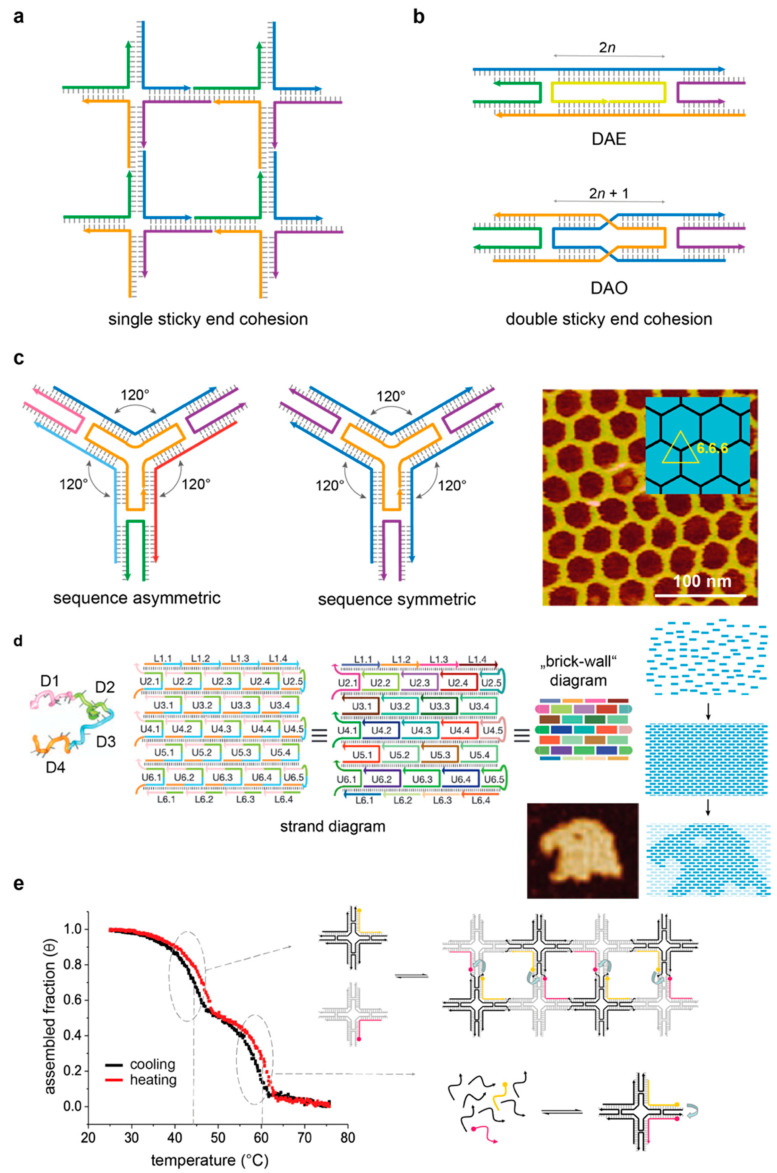
Multi-stranded approach. (**a**) Single sticky-end cohesion between four-arm junctions enables the creation of stick-like objects, where edges are double-helical stretches and vertices are branched points. (**b**) A branched DNA motif containing two crossovers is the (double-crossover) DX tile. The most used isomers are the DAE and DAO tiles. Both double-crossovers (D) display antiparallel (A) helical domains and, respectively, an even (E) and odd (O) number of half-helical turns in between the two crossovers. (**c**) A three-point star comprised of three four-arm junctions merged at a common end. The sequence identity of the component strands enables the generation of asymmetric (left panel) or symmetric (middle panel) motifs. Both can grow along three directions, tiling the plane in a 6.6.6 pattern (right panel). Reprinted with permission from reference [49], Copyright 2005, ACS. (**d**) Extending the sequence asymmetry principle to the simplest form of tile leads to the single-stranded tile (SST) or brick approach. The method relies on the use of several hundreds of distinct sequences designed to hybridize one another into simply concatenated duplexes, thus giving rise to 2D or 3D structures with a high level of complexity and addressability. Reprinted with permission from reference [50], Copyright 2012, Springer Nature. (**e**) Thermal-dependent fluorescence resonance energy transfer (FRET) spectroscopy can be applied to monitor in real time the assembly and disassembly of single tiles and, thereof, lattices. Reprinted with permission from reference [51], Copyright 2009, Wiley and Sons.

Four-arm junctions are not the only branched DNA molecules that have been realized and explored. Applying a strict principle of sequence-symmetry minimization [35,36], three-arm, as well as five-, six- and twelve-arm junctions [37,38,39], have been constructed and characterized. According to this principle, the sequences participating in the formation of the motif are chosen to be maximally different one from the other in order to minimize the competitive formation of alternative structures. The next level of sophistication entails the use of such branched DNA molecules for the construction of geometrical objects, where the edges are double-helical stretches and the vertexes are branch points (Figure 3a). This can be achieved by prolonging the arms of the junctions with single-stranded sticky ends in order to direct the self-assembly of the tile units into stick figures, such as a quadrilateral [40] or a cube [41] or large periodic arrays [42]. A sticky end is a short stretch of single-stranded DNA that extends from the 5′ or 3′ end of a double-helical region and allows for the hybridization to a complementary sticky end from a distinct duplex. Unfortunately, the structures resulting from cohesion of branched junctions are rather flexible and, therefore, cannot function as true crystals. This problem was solved with the introduction of more complex and structurally constrained tiles formed by merging multiple branch junctions together, as discussed in detail below.

### 4.1. Multi-Branched DNA Tiles

Typical multi-branched DNA motifs are double crossovers (DX) [43,44], triple crossovers (TX) [45], DNA parallelograms [46] and four-by-four structures (also called four-point stars) [47]. Such constructs are characterized by multiple branch points connected into larger and stiffer tiles. The smallest member of this class is the DX tile [43], which is composed of two double-helical domains that cross each other in two points (Figure 3b). Five isomers of the DX molecule can be distinguished (DAE, DAO, DPE, DPOW and DPON) on the basis of the relative orientation of the helical domains (Antiparallel or Parallel), as well as the number of half-helical turns (Even or Odd) that separates the two crossovers of the tile and the insertion of the last of an odd number of half-turns into a major (Wider) or minor (Narrow) groove. All DX tiles, however, share a common feature—that is, a linear geometry with two pairs of sticky ends pointing in one direction and two pairs of sticky ends pointing in the opposite direction of the same motif. The system therefore assembles with double sticky-ended intermolecular interactions instead of single sticky ends, thus making the connections more rigid and less susceptible to individual variations [48]. 

An interesting aspect of multi-branched tiles is their symmetry (both in terms of sequence and topology) and how this affects the outcome of the assembly process. Typically, a multi-branched DNA tile is composed of a distinct number (i) of arms, with each arm equal to a four-way junction. As the arms originate from a central branching point and are oriented radially, the motifs are referred to as i-point stars. A DX tile could be in principle viewed as a two-point star, with two four-arm junctions pointing in opposite directions. To illustrate the symmetry aspect of a multi-branched motif, it is more convenient to consider the three-point star, though the same reasoning applies also to tiles of higher order. The three-point star has been employed as the building unit of several DNA objects following one of two possible design strategies. These strategies differ in the sequence symmetry of the tile—that is, in the number of distinct oligonucleotides used for its assembly (Figure 3c). In a first “asymmetric” approach, directly derived by the sequence-symmetry minimization principle proposed by Seeman, each arm of the tile is a distinct four-arm junction [52]. Merging three such arms into the star motif results in a total of seven distinct strands: one long central strand, three lateral strands of medium length and three short strands (Figure 3c, left panel). In a second “symmetric” approach, initially developed by Mao [49,53,54], the same topology is achieved by using only three distinct sequences resulting from a three-fold repetition of the same four-arm junction (Figure 3c, middle panel). Thus, despite the fact that the two objects display the same geometry and can grow along three directions, the self-assembly of the asymmetric motif can be controlled at the level of each single arm-to-arm linkage, whereas the degeneracy of the symmetric tile will result in the statistically equivalent (and, therefore, indistinguishable) linkages along each of the three directions. Sequence symmetry and shape symmetry of the final assembly product are therefore not related by a one-to-one correspondence. Indeed, whereas a tile that is symmetric in terms of sequence necessarily leads to a geometrically symmetric object, the opposite is not necessarily true, meaning that a structure that is symmetric in shape (i.e., has an i-fold symmetry) does not need to be symmetric in the sequence of its component strands.

### 4.2. Structural Properties of Multi-Stranded Nanostructures

The initial vision and final goal of structural DNA nanotechnology is to construct molecular architectures of desired geometrical features. Thus, once suitable junctions are realized, the next step is to connect them into networks. As mentioned above, this is done mostly through double sticky-end cohesion and consists in the hybridization of complementary single-stranded segments protruding from the 5′ or 3′ termini of distinct junctions’ arms. The base sequences of the sticky ends essentially define the connectivity between tiles, i.e., how tiles are linked to one another in the final object. On the other hand, the final geometry of the self-assembled product is encoded in two additional features of the system: (i) the angles between the arms of each tile and (ii) the distance between the joined crossovers of two adjacent tiles. The angles at the center of a multi-branched tile are normally defined by the length of the thymine loops inserted between adjacent arms and by the total number of arms among which the mechanical stress of the motif is distributed. For example, inserting three thymine bases between two adjacent arms of a three-point star leads to a motif with a C3 rotational symmetry, which, upon self-assembly, tiles the plane into a regular hexagonal pattern (Figure 3c, right panel). Such a tiling is also referred to as 6.6.6, indicating that, at each junction, three hexagons meet at their vertices, tiling the plane into three identical angles of 120° [49,55]. By lowering the sequence symmetry of the system, distinct Archimedean tilings can be obtained [52]. Starting, for example, from the same three-point star, the number of thymine loops at the center of the motif can be varied such to result into one angle of 90° and two angles of 135°. Such a motif will tile the plane into regular squares and octagons in a 4.8.8 pattern. Similarly, using a four-by-four tile, regular square-like patterns (4.4.4.4) or 3.6.3.6 tessellations can be achieved.

One should also consider that the torsional stress at the center of a multi-branched tile gives rise to dihedral rather than planar angles, thus causing the tile to bend. Such an intrinsic curvature is of fundamental importance for the final architecture of the self-assembly product. Indeed, completely different geometries can be obtained depending on the other parameter of the tile-to-tile association, i.e., the distance between the crossovers of two adjacent tiles. Considering *n* as the number of half-helical turns between joined crossovers, an even multiple of half-helical turns (2*n*) will result in accumulation of the intrinsic curvature of the tile and formation of a closed 3D assembly [54]. On the contrary, cohesion of adjacent tiles through an odd multiple of half-helical turns (2*n* + 1) will cause them to face up and down alternately (according to a so-called “corrugation strategy”), with formation of an open quasi-planar array [47]. 

A final consideration applies to the relationship between the periodicity of the assembly and the sequence symmetry of the building unit. A periodic array of the type A*_n_* will result from the self-association of a single tile (A) along all possible growing directions and will require the sticky ends to be either pairwise complementary (for an even number of arms) or self-complementary (i.e., palindromic, for an even or odd number of arms) [49,56]. If the target object is a lattice of the type (AB)*_n_*—that is, a periodic array of two distinct tiles, A and B—mutually complementary cohesions must be instead designed [57,58]. Increasing the number of distinct tiles that participate to the assembly reaction means to decrease the sequence symmetry of the system, which, in turn, prevents the assembly of periodic structures of unlimited size. On the other hand, the use of multiple tile connections with a large number of distinct strands enables the realization of molecular pegboards that display many different and singularly addressable positions [59,60]. Thus, whereas the sequence symmetry of the building unit facilitates the formation of large and periodic arrays, sequence asymmetry is the route to achieve higher complexity. In 2012, Yin and coworkers developed a design strategy that extrapolates this concept to the simplest form of tile: a single-stranded DNA (therefore, called the single-stranded tile or SST approach) [50] (Figure 3d). The tile consists of 42 bases divided into four domains, each binding to four local neighboring tiles during self-assembly. Considering each SST as a pixel, the method is based on the generation of a master strand collection that corresponds to a more than 300-pixel canvas, from which appropriate subsets of strands are selected to achieve a desired two-dimensional shape. The same principle has been extended to three-dimensional structures, using so-called “DNA bricks” as building blocks of 3D canvas [61]. The SST and bricks assembly methods thus provide a modular framework for constructing aperiodic nanostructures with a high level of complexity.

### 4.3. On the Thermal Assembly of Multi-Branched DNA Tiles and Their Extended Nanostructures

While the thermal folding/unfolding of the Holliday junction has been extensively characterized, less has been done on multi-branched DNA tiles and derived arrays. The melting curves of DNA complexes provide a measure of the thermal stability and cooperativity of intermolecular interactions, which are indicated, respectively, by the temperature at the transition midpoint (*T*_m_) and the width of the transition [62]. Initial UV spectroscopy studies on the DAO motif showed a monophasic cooperative melting profile around 60 °C, almost 20 °C less than the corresponding duplexes building the same motif [63]. Interestingly, triple crossovers (TX) with similar base compositions melt at approximatively the same temperature as the corresponding DX tiles but show a biphasic profile in which two transitions are visible, with the second being less pronounced and centered at a higher temperature (ca. 75 °C) [45]. This result indicates that DNA tiles, particularly when large in size and complex in topology, may display domains with distinct thermal properties and an energy profile with multiple transitions. Another example of such a trend is the paranemic motif (PX) [64]. In a PX tile, two double strands are wrapped about each other similarly to the two strands of a double helix, leading to a series of contiguous crossovers at each possible site of strand exchange. The melting curves of PX molecules indicate a cooperative process and a large degree of hysteresis even at extremely slow heating rates. In this case, too, the thermal transitions are biphasic, with an initial pre-melting disruption of the PX structure, followed by full unstacking of the nucleotides. Such systems cannot be idealized as two-state models, calling for alternative approaches that enable the reliable analysis of the data and extrapolation of the thermodynamic parameters of the thermal transition.

A few years later, Saccà and coworkers applied a thermal-dependent FRET spectroscopy method to investigate the thermal properties of selected regions of a four-by-four tile [65]. Contrary to the UV-based approach that gives information on the global thermal stability of the construct, the FRET-dependent method is indicative of the region nearby the two fluorophores and provides a quantitative measurement of their relative distance (within a range of 1 to 10 nm). By positioning the two fluorophores at variable locations of the tile, it was possible to reveal the existence of tile domains characterized by distinct melting temperatures and free energies of transition. When applied to the assembly/disassembly of a periodic lattice of two distinct four-by-four tiles, the method enabled the identification of two well-separated transitions: a first transition at a high temperature (ca. 60 °C) corresponding to formation of the tile and a second transition at a low temperature (ca. 40 °C) associated to tile-to-tile cohesion [51] (Figure 3e).

The thermal stability of tile-to-tile interaction is directly related to the strength of the sticky-ends cohesion. This can be typically enhanced by increasing the GC ratio and/or the length of the complementary DNA segments. On the other hand, excessive hybridization forces should be avoided, as this might result in the trapping of misfolded structures, thus impairing the formation of the desired network. As a rule of thumb, three-to-five nucleobase-long sticky ends are normally sufficient to ensure specificity and stability of the newly formed double-helical bond, while enabling self-correction mechanisms to take place.

## 5. The Scaffold-Based Approach

In the previous section, we described the structural and thermal properties of multi-stranded DNA objects, historically the first type of structures established in the field of DNA nanotechnology [3]. An extraordinary breakthrough in the construction of nanometer-sized DNA objects occurred in 2006 with the introduction of the scaffolded DNA origami method by Paul Rothemund [5] (for reviews on this subject, see, for example, [8,9,10]). Similar to the Japanese art of paper folding, the DNA origami technique relies on the discontinuous hybridization of a long single-stranded DNA (termed scaffold) to a few hundreds of short oligonucleotides (called staple strands). These latter are computer-designed, such that the outcome of the self-assembly has a desired shape. Contrarily to the multi-stranded method, the origami approach needs a long template sequence as the “main actor” of the assembly process. Whereas, in the multi-stranded approach, all strands mutually interact one with the other; in the DNA origami method, several staple strands hybridize to a single scaffold rather than with each other. This fundamental difference has a notable impact on the yield of product formation. The multi-stranded method requires that the relative stoichiometry and purity of the interacting strands are carefully controlled and adjusted, resulting in error-prone and lengthy synthetic processes. By contrast, the presence of a scaffold in the origami method ensures that the initial correct arrangement of the first strands favors the further binding of the remaining staples, such that their stoichiometric ratio and purity grade are no more relevant, with obvious advantages in terms of assembly time and efficiency. A deeper discussion on the proposed mechanisms of DNA origami assembly compared to the multi-stranded approach will be given in Section 6.

### 5.1. Structural Properties of Scaffold-Based Assemblies 

**Figure 4:**
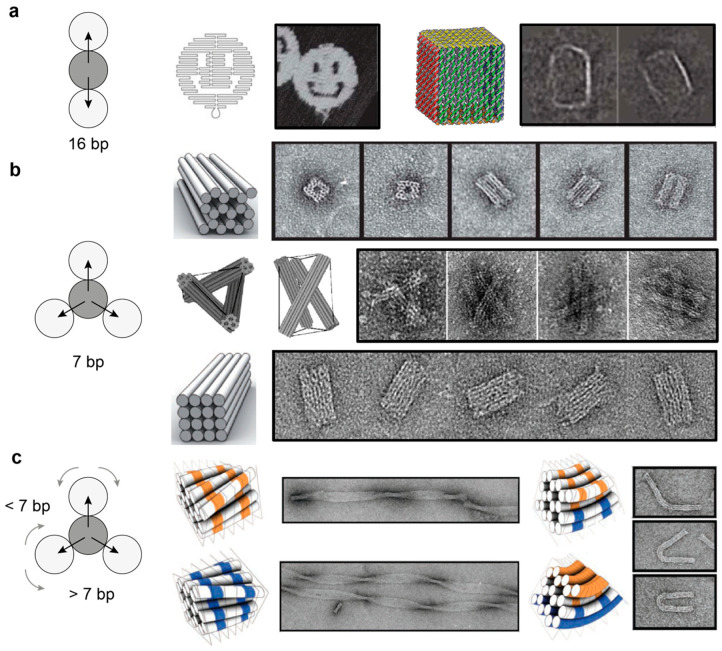

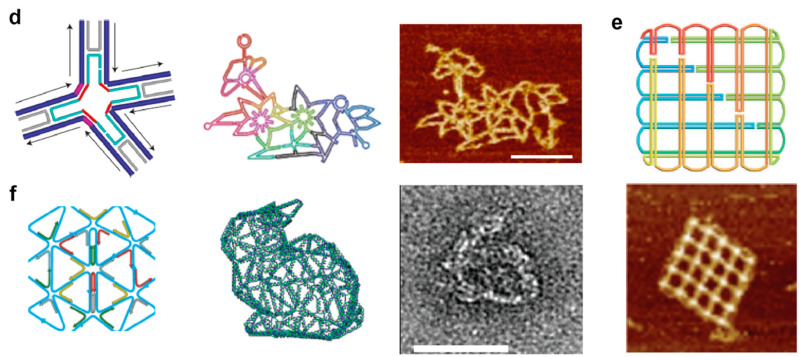
Scaffold-based approach. Single-layer (**a**) and multi-layer (**b**,**c**) DNA origami structures can be generated by the suitable engineering of crossovers, thus leading to a large variety of shapes, including vacant, filled, twisted and curved objects. DNA helices are indicated by circles and are viewed along their central axis. Interhelical crossovers between a reference helix (grey circle) and its neighboring helices (white circles) are indicated as black arrows. The number of base pairs between consecutive crossovers on antiparallel connected helices is also given. Reprinted with permission from reference [5], Copyright 2006, Springer Nature; [6], Copyright 2009, Springer Nature; [7], Copyright 2009, AAAS; [67], Copyright 2009, Springer Nature; [72], Copyright 2010, Springer Nature. (**d**–**f**) The same principle is applied to multi-arm junctions with arbitrarily designed connections, allowing for the construction of sophisticated 2D and 3D patterns. Reprinted with permission from reference [73,74], Copyright 2015, Springer Nature; [75], Copyright 2016, Wiley and Sons.

The structural properties of DNA origami architectures have been extensively reviewed in the past few years (as an example, see [8,9,10]). We will therefore keep this section intentionally short and invite the interested reader to refer to the vast literature available on this topic. Briefly, the final shape of a DNA origami object is given by the arrangement of crossovers between the connected helices. In the original work by Rothemund [5], several planar origami structures were produced, ranging from simple rectangular shapes to more complex forms such as stars, triangles, and smiley faces. These objects were generated by following a relatively simple design rule: every helix within the structure is connected to two neighboring helices by a regular pattern of crossovers interspaced by 1.5 helical turns, which for B-type DNA, correspond to about 16 base pairs (Figure 4a). This register of crossovers generates interhelical connections every 180°, leading to a single layer of helices arranged into a planar sheet. The same design principle—however, with distinct crossover patterns—can also be used to generate three-dimensional structures formed by planar sheets connected into prism-like architectures [66,67,68,69,70] or by multiple helices packed together into space-filled objects with different curvatures and twists [6,7,71] or connected through compression and tensile strengths, according to the well-defined tensegrity rules [72] (Figure 4b,c). Finally, and of particular note, are the so-called scaffold-based wireframe structures. Different from the canonical origami structures, in which the helices are organized into parallel raster-fill patterns of honeycomb or square-lattice geometry, wireframe architectures are realized by routing the scaffold through multi-arm junction units that are linked together by one or two double-helical stretches. As each junction may contain from 2 to 12 arms, their mutual connection allows for the nonparallel alignment of several DNA helices. This eventually results in the formation of 2D net-like surfaces with various sizes and shaped cavities, as well as 3D polyhedrons, curved solids and multi-layered frameworks [73,74,75] (Figure 4d–f).

### 5.2. Thermal Assembly and Disassembly of DNA Origami Structures

Once the realization of DNA origami structures became feasible for many laboratories around the world, the interest of many scientists in the field moved to the underlying theoretical questions behind the process: “How does DNA origami self-assembly actually occur?” and “How is it possible that hundreds of different components associate together into a unique structure, most often in a few hours and—most surprisingly—in very high yields, apparently against all reasonable expectations?” A similar structural problem is encountered in protein folding. In favorable conditions, protein folding occurs spontaneously and in a relatively short time; however, the target structure must be selected from an exponentially large conformational space and clearly cannot be the result of a random search. A possible solution to this paradox is captured by the so-called principle of “minimal frustration” [76], according to which, the folding pathway of a protein towards its most probable configuration is biased by a rapid gain in energy stabilization for conformations progressively similar to the native state. When applied to nucleic acids, free energy landscape formalisms have provided the conceptual frameworks to describe the thermally or mechanically induced transitions of Holliday junctions [28,34], DNA hairpins [77,78,79] and G-quadruplexes [80]. More complex DNA structures, such as, for example, a DNA origami, must therefore obey a similar rule, with the primary sequence, i.e., the set of staple/scaffold base-pairing interactions, encoding the information for folding.

**Figure 5:**
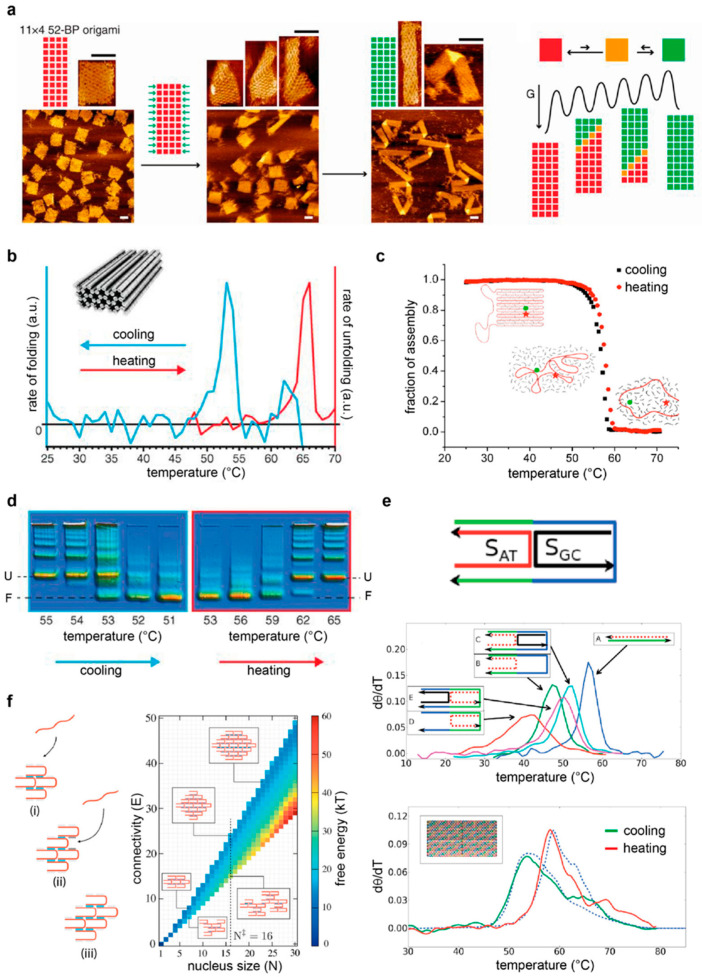
Experimental observations and theoretical models of DNA nanostructures’ assembly. (**a**) The structural reconfiguration of a DNA origami structure from one state (red) to another (green) is mechanically triggered by the addition of staples that target the edges of the shape, passing through intermediate species (orange) along a folding landscape. Reprinted with permission from reference [85], Copyright 2017, AAAS. The folding and unfolding of templated DNA objects can be monitored fluorometrically at the global (**b**) or local (**c**) level. Reprinted with permission from reference [89], Copyright 2012, AAAS; [90], Copyright 2013, ACS. Whereas the former method emphasizes the hysteresis of the transition, the latter method is more suitable to identifying the conformational domains of the structure. In both cases, however, the transition is cooperative. (**d**) Gel electrophoresis analysis of the reaction mixture at different time points evidences the progressive formation (or disruption) of the target shape with the temperature. (**e**) Minimal origami model representing the connectivity of the staples in the presence of a scaffold. Upper panel: the AT-rich sequence (S_AT_) is in the outer position, and the GC-rich sequence (S_GC_) is in the inner position. Middle panel: UV profiles for the folding of S_AT_ in presence or absence of S_GC_. Lower panel: annealing (green) and melting (red) of a DNA planar structure and corresponding simulations (blue) of the model. Reprinted with permission from reference [88], Copyright 2013, AIP. (**f**) Model of cooperative growth for an SST structure. Each tile binds through a single binding domain (i), creating two available binding sites at the interface (ii) that, once occupied by a second tile, lead to extra stabilization effects (iii). Free-energy landscape for assembly of the SST structure as a function of the size (*N*) and number of bonded domains (*E*) between tiles. Reprinted with permission from reference [94], Copyright 2018, AIP.

Important progress along these lines has been made by monitoring the outcome of the assembly reaction under different experimental conditions or visualizing the formation of intermediate species at different time points of the thermal annealing or melting process. Atomic force microscopy (AFM) studies have been particularly informative, as they can provide structural details at the single-particle level and have demonstrated the existence of distinct folding pathways [81,82,83,84,85,86,87] (Figure 5a). Spectroscopic methods have instead been mostly employed to gain quantitative information on the free energy associated to the construction or disruption of the origami structure, either at the global level (using UV-Vis absorbance or fluorometric methods) [88,89] (Figure 5b) or restricted to local regions around selected strands (FRET spectroscopy) [87,90] (Figure 5c). Although the majority of these studies rely on the use of a temperature gradient for association or dissociation of a DNA origami structure, both agarose gel electrophoresis and AFM studies demonstrated that correct folding can be achieved at a defined and constant temperature, i.e., isothermally [89] (Figure 5d). Differently from thermal experiments, which give access to the thermodynamic parameters of the process, isothermal data may provide information on the kinetics of the reaction and, thus, are more prone to identifying the sequential order of events that take place during the self-assembly within the time of observation [91].

The wealth of data collected until now on the assembly/disassembly of a DNA origami structure can be distilled into three main points. First, the formation and the disruption of a DNA origami structure are characterized by a certain degree of hysteresis. This means that the process of assembly and disassembly are not reversible, i.e., the two processes travel two different paths of the energy landscape. A way to characterize the degree of hysteresis, i.e., the extent of irreversibility of the transition, is to indicate the difference between the temperature at the mid-point of the transition, *T*_m_, for each of the two processes. The higher this difference, the greater the hysteresis experienced by the system. Each transition, however, takes place in a relatively short interval of temperature around the *T*_m_, reflecting the strong cooperativity of the process. This second aspect has been often associated to the tight connectivity of the strands that constitute the structure. In other words, the strong mechanical coupling between the building units of an origami structure would result in a collective thermal behavior, once a discrete number of entangled strands undergoes a thermal transition. The extent of hysteresis and cooperativity may be modulated by the initial conditions of the assembly reaction, thus leading to the third and final feature of DNA origami constructs, which is the plasticity of the folding path. Indeed, a change in the connectivity of the strands (and, therefore, in the topology of the building motifs) will reflect in a change of folding/unfolding behaviors. In some cases, this may result in the formation of a completely different structure as the consequence of an allosteric propagation of a mechanical transformation from one initial site to the entire set of coupled junctions (Figure 5a). Such a phenomenon has been observed either upon application of an external mechanical load (such as a stretching force [83]) or upon the addition of so-called “trigger” strands. These triggers typically target the edges of the origami structure, i.e., those exposed regions of the structure where the scaffold inverts its direction, with a direct impact on the entropic penalty of scaffold bending [85,86,87]. Besides a topology-dependent entropic effect, the outcome of an assembly reaction may be also affected by an enthalpic contribution related to the sequence content of the component strands. Intuitively, high melting-temperature (GC-rich) sequences will participate in the folding process at an early stage and will thus play a dominant role in guiding the process, as compared to AT-rich sequences. In other words, while the connectivity of the strands will affect the kind of topological transformation occurring, their base sequences will determine if this transformation constitutes one of the key guiding events of the self-assembly process from which folding will proceed. The basic concept is therefore to identify any possible relationship between the nucleation sites and the topologically frustrated sites [87]. Finally, a further contribution to the folding pathway undoubtedly comes from the environmental conditions of assembly, including the temperature at which the reaction takes place, as well as the type and concentration of salts in the reaction buffer. Recent studies show that the beneficial effect of magnesium ions is largely dependent on the DNA superstructure and that, in some cases, those structures can survive in almost pure water for long times [92]. Only a few examples of DNA nanostructures have been reported in the presence of alternative buffers, such as 4-(2-hydroxyethyl)-1-piperazineethanesulfonic acid (HEPES) [68], cacodylate [93] or phosphate-buffered saline (PBS) [74].

## 6. Proposed Models of DNA Nanostructures’ Assembly

We dedicate this final section to a comparative analysis of the two main DNA design schools, the multi-stranded and the scaffold-based approach, with the purpose to discuss the general guiding principles of complex DNA nanostructures’ assembly. As briefly mentioned above, the main difference between these two approaches relies on the presence or the absence of a template, i.e., a long single-stranded DNA chain that guides the folding of the DNA object. In simple terms, the role of the scaffold is to physically connect the component strands, establishing long-range interactions that bring them spatially closer one to the other. This effect is, of course, more evident at the edges of the structure, where the scaffold inverts its direction and forms a loop. 

To better understand the implications of a guiding template, Arbona and coworkers developed a minimalist origami model that cleverly recapitulates the essential features of a scaffold-based approach [88,95]. The structure under investigation, referred to as mini-origami, consists of three DNA strands: two short, staple strands, respectively called the outer and inner strands, that bind to two discontinuous or adjacent regions of a third, longer strand, which serves as a scaffold (Figure 5e, top panel). By changing different parameters of the system that selectively address the thermal stability of the single strands, as well as their connectivity and cooperativity, the authors succeeded in generating a model that accounts for both the topological constraint (Δ*G*_top_) and the free energy of the hybridization (Δ*G*_NN_) of the connected staples and matches the experimental data of large planar DNA origami objects with surprising precision (Figure 5e, lower panel). The main point of this model is to realize the importance of the scaffold crossovers and to quantify their impact on the self-assembly process. Indeed, whereas staple crossovers are a necessary feature of all DNA nanostructures, as they ensure the intertwining of distinct double helices into the target DNA object, scaffold crossovers, i.e., points of scaffold route inversion, are a prerogative of template-assisted methods. Thus, when two adjacent helices share the same scaffold strand, the addition of an outer staple generates a loop between the two discontinuous regions of hybridization. The longer this loop, the lower the probability of these regions to be close to each other and, thus, the lower the probability of the outer strand to hybridize to both complementary stretches simultaneously. This translates into an entropic penalty that is proportional to the length of the loop. However, when an inner strand is present that binds and bends the loop region, pairing of the outer staple is facilitated, meaning that the entropic penalty will decrease. This mechanism has been clearly demonstrated by UV melting studies and powerfully highlights the concept of topological connectivity between the staples of an origami-like motif (Figure 5e, middle panel). Such a topology-dependent contribution (Δ*G*_top_) is responsible for the cooperativity of the thermal transition and is accounted for at the local scale, introducing correlations between the probability of the staples to share the same neighborhood. Clearly, which of the two strands, the outer or the inner, will first bind to the scaffold strand will strongly depend on their thermal stabilities, i.e., ultimately, on their GC content. In other words, the chemical sequence of the strands will define their hierarchical correlations, with a strong impact on the temporal order of events that occur during the annealing and melting of the structure. This sequence-dependent contribution to the local formation of double helices is quantified using the parameters of the nearest-neighbor model (Δ*G*_NN_). Thus, assuming a local equilibrium for the insertion process of the staples, the model of Arbona shows that the folding of each strand depends on the presence of a nearby cluster of folded strands according to both topologically dependent entropic restraints and sequence-related enthalpic contributions to double-stranded DNA (dsDNA) hybridization. The nonequilibrium aspects (such as hysteresis) are therefore explained by the fact that the neighborhood of the staples is different in the annealing and melting process. Other theories have been advanced that build on this minimalist approach and consider additional contributions to explain the observed cooperativity of DNA origami folding, as well as the asymmetry of its hysteresis. Such contributions include the coaxial stacking of adjacent domains and Kuhn lengths approximations of the loop, as well as a conformational entropy and binding enthalpy that affect the step-wise folding of DNA origami structures in a loop’s length-dependent fashion [96,97].

Whereas the mini-origami model essentially relies on the formation of loops between adjacent connected double helices, an appropriate model of the multi-stranded approach should instead exclude any contribution from scaffold loops and consider only the connectivity of the strands that build up the target shape. One such system, and probably the most representative of a true multi-stranded approach, is the SST (or brick) assembly. Here, structures are kept in place only through a pattern of staple crossovers that join adjacent antiparallel helices in the absence of any scaffold strands. The formidable complexity of such structures has been investigated in several theoretical studies [94,98,99,100,101], due to the surprising rate of successful assembly despite the huge number of component strands. Initial Monte Carlo simulations of such systems were performed, idealizing the single-stranded molecules as particles with four tetrahedrally arranged patches, with each of these patches corresponding to a specific DNA sequence [99]. The results showed the existence of a free-energy barrier to nucleation that increases with the temperature. At high temperatures, nucleation events become rare; at lower temperatures, by contrast, several nuclei can form simultaneously and, subsequently, form large aggregates. Self-assembly of the target structure can therefore successfully take place only within a small window of temperatures, which ensures that the nucleation barrier is low enough to be crossable on experimental time scales but sufficiently high to allow a time separation between consecutive rounds of nucleation and growth, thus suppressing misfolding and aggregation. The study revealed that such a particular requirement can be met, regardless of the choice of the sequences, by using a slow annealing process that starts from high temperatures. In these conditions, the system will always pass through the optimal nucleation regime during cooling and will spend there a sufficient amount of time to enable fast and effective growth. The temperature ramp during the assembly protocol is therefore, here, much more critical than in other DNA design approaches, and a random choice of sequences’ bond energies seems to be even more effective than a homogeneous distribution of the hybridization interactions [100]. Later, the same authors used an off-lattice model [101] to account for the translational and rotational entropy of the single strands, being able to reproduce the main physical phenomena observed in the simpler lattice model. A recent study instead applied oxDNA [102] to calculate the thermodynamics of the tile association. Contrary to previous patchy-particle models (that rely on the broken symmetry of the building units to explain the directional self-assembly behavior), this model describes explicitly the polymeric degrees of freedom of DNA, thus making it more suitable to capture the entropic penalties associated with the binding of successive domains during tile association [94] (Figure 5f). Despite their subtle theoretical differences, all SST models emphasize the importance of an accurate temperature ramp protocol: only in this way can the system cross the nucleation barrier to reach on-pathway species that are necessary to avoid misfolding and aggregation. Upon further cooling, partially formed assemblies can finally stabilize to the full target product. In order to find the optimal temperature for error-free assembly, DNA brick constructs are usually cooled over several days in a very narrow temperature range or isothermally assembled slightly above the average melting temperature of the dsDNA segments. The assembly can be further modulated by the length of the dsDNA segments, their GC contents or splitting them into segments; alternatively, the salinity of the buffer, as well as the addition of molecular crowding agents, can be considered [103].

## 7. Conclusions

It may be tempting to view the two different strategies of DNA nanostructures’ assembly, the multi-stranded and the scaffold-based approach, as two faces of the same coin, i.e., as two strategies that, although extremely different, highlight two opposite aspects of a general nucleation-and-growth mechanism. The interpretation of DNA nanostructures’ assembly using the classical nucleation theory typical of crystals’ growth is, however, still a matter of debate in the field. Theoretically different approaches have led to models of SST and bricks assembly that are equally suitable to describe the experimental observations, although their interpretations of the data completely diverge in terms of applicability of the classical nucleation theory [94,100,101]. A similar argument appears when describing the assembly of a DNA origami. Here, staples hybridize to the scaffold and not to the other staples, as in the SST approach, thus raising questions on the correctness of the term “nucleation” to describe the establishment of the initial binding events in such systems [96]. Despite such subtle controversies, the constant refinement of the theoretical models greatly improves our capability to reproduce and predict experimental outcomes and brings us now to the next challenge: Are there alternative ways to guide DNA self-assembly?

**Figure 6:**
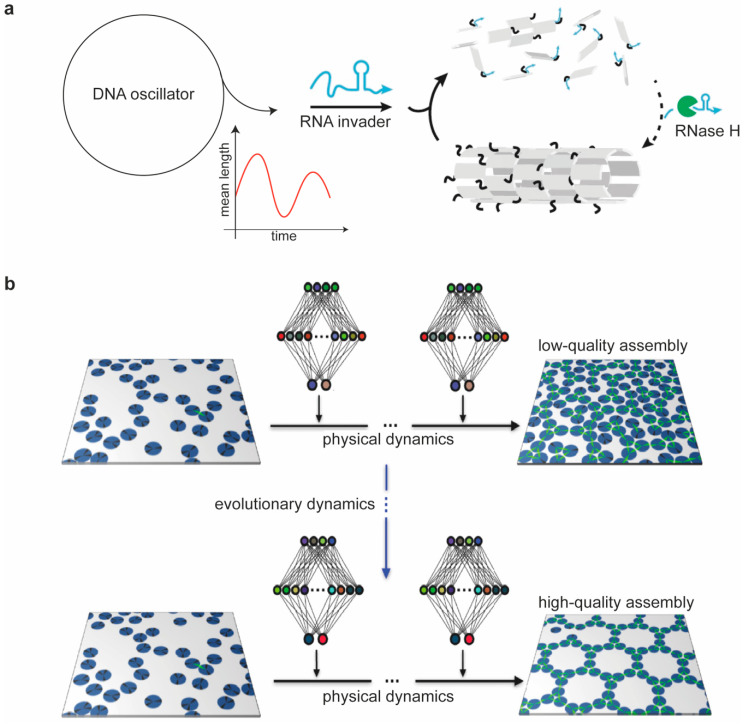
Alternative strategies for the autonomous self-assembly of DNA nanostructures. (**a**) A synthetic autonomous oscillator based on DNA reaction cascades is used to direct the disassembly of DNA nanotubes through RNA invader strands. These bind to the nanotube tiles and cause them to dissociate. In the presence of RNase H, the RNA invader strands bound to the tiles are degraded, thus allowing them to reassemble into the original nanotube structure. Consecutive cycles of RNA invader strand production and RNase degradation result in the oscillating dynamic behavior of the system. Reprinted with permission from reference [104], Copyright 2019, Springer Nature. (**b**) Neural network policies trained by evolutionary reinforcement learning can enact efficient time- and configuration-dependent protocols for molecular self-assembly, guiding the formation of desired structures or polymorphs. Reprinted with permission from reference [105], Copyright 2020, APS.

The answer to this question is surely affirmative. In the past 40 years, since the very beginning of structural DNA nanotechnology, we witnessed an exciting development of DNA design strategies and assembly protocols, some of which apparently against any realistic possibility of success and, nevertheless, surprisingly effective. The field is therefore still open to further exploration and may probably still hold many surprises that are worth investigating. Along this line, an emerging area of interest is represented by synthetic self-assembly systems that are driven and maintained in out-of-equilibrium conditions. Being capable of autonomous life-like behavior, such DNA-based materials can thus truly emulate the building principles of more complex biological entities. A relevant example of this kind was recently shown by Franco and coworkers [104]. In their work, the disassembly and formation of DNA nanotubes was controlled, respectively, by the addition of RNA invader strands and their digestion by RNase H (Figure 6a). When the invaders bind to the nanotubes’ tiles, it causes them to disassemble. In the presence of RNase H, the RNA invader strands bound to the tiles are degraded, thereby making it possible for tiles to reassemble. The extent of RNA invasion is, in turn, regulated through an autonomous molecular oscillator constituted by several DNA cascade reactions connected into a cyclic pathway. The continuous replenishment of invader strands keeps the process in a nonequilibrium state and results in large-amplitude oscillations of the nanotubes’ lengths as a consequence of alternating growth and degradation events. Capitalizing on previous works on reconfigurable DNA nanostructures, this study represents one of the most complex examples of a successful coupling of a nonlinear dynamic system with a downstream growing pathway. Another recent example instead tackles a conceptual challenge of molecular self-assembly—that is, our limited theoretical understanding of what actually occurs during the process. Indeed, although the final outcome of the reaction is most often a species at the equilibrium regime, the reaction itself happens in out-of-equilibrium conditions, and the application of theoretical models to decipher the path is either not fully appropriate or requires approximations of local equilibrium states. Whitelam and Tamblyn recently proposed an alternative approach based on machine learning to face this problem [105]. The method seeks to control self-assembly without human intervention beyond the specification of which parameters of the system need to be promoted. Most importantly, the algorithm works without prior understanding of what may constitute a “good” assembly protocol. Using molecular simulations, the authors showed that neural networks trained by evolutionary reinforcement learning can control self-assembly protocols, enhancing the survival of desired structures over possible competitors, thus opening the way to the design and optimization of assembly protocols by artificial intelligence (Figure 6b).

Thus, from the discovery of its structure almost 70 years ago, the DNA molecule has shown us the enormous possibilities held in the simplicity of the Watson-Crick base pairing rule, enabling us to construct, reshape and link structural modules into systems of increasing structural complexity and intriguing dynamic behavior. The next challenge appears to be the creation of self-organizing systems with emergent properties, i.e., capable of emulating a true evolutionary process through progressively fittest species. This, however, will require a change of paradigm, from thermodynamic to kinetic control, from equilibrium to out-of-equilibrium conditions and from reversible to irreversible changes. The challenge is certainly ambitious, but initial efforts in this direction have already shown us that this is possible.
